# Simulating Vapor–Liquid–Solid Growth of Au-Seeded InGaAs Nanowires

**DOI:** 10.1021/acsnanoscienceau.1c00052

**Published:** 2022-02-07

**Authors:** Erik K. Mårtensson, Jonas Johansson, Kimberly A. Dick

**Affiliations:** † NanoLund and Division of Solid State Physics, Lund University, SE-221 00 Lund, Sweden; ‡ Centre for Analysis and Synthesis, Lund University, SE-221 00 Lund, Sweden

**Keywords:** III−V, Nanowire, Ternary, Simulation, Monte Carlo, InGaAs, Au

## Abstract

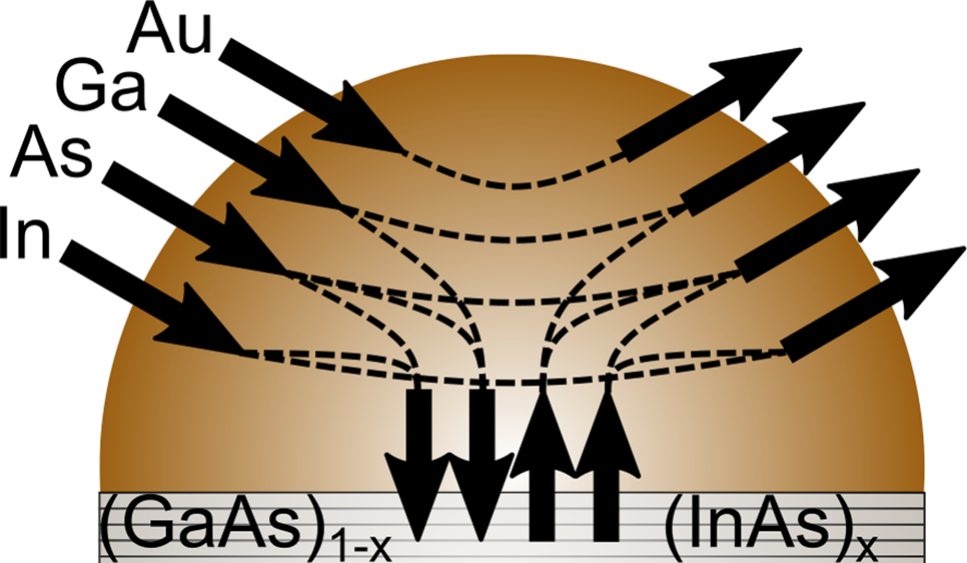

Ternary III–V nanowires are commonly grown using the Au-seeded vapor–liquid–solid method, wherein the solid nanowires are grown from nanoscale liquid seed particles, which are supplied with growth species from the surrounding vapor phase. A result of the small size of these seed particles is that their composition can vary significantly during the cyclical layer-by-layer growth, despite experiencing a constant pressure of growth species from the surrounding vapor phase. Variations in the seed particle composition can greatly affect the solid nanowire composition, and these cyclical dynamics are poorly understood for ternary nanowire growth. Here, we present a method for simulating nanowire growth which captures the complex cyclical dynamics using a kinetic Monte Carlo framework. In the framework, a nanowire grows through the attachment or detachment of one III–V pair at the time, with rates that are based on the momentary composition of the seed particle. The composition of the seed evolves through the attachment and detachment of III–V pairs to the solid nanowire and through the impingement or evaporation of single atoms to the surrounding vapor. Here, we implement this framework using the As–Au–Ga–In materials system and use it to simulate the growth of Au-seeded InGaAs nanowires with an average solid Ga/III ratio around 0.5. The results show that nucleation preferentially occurs via clusters of InAs and that the compositional hierarchy of the liquid seed (*X*_As_ < *X*_Ga_ < *X*_In_) determines much of the dynamics of the system. We see that imposing a constraint on the simulation, that only the most recently attached III–V pair can be detached, resulted in a significant narrowing of the compositional profile of the nanowire. In addition, our results suggest that, for ternary systems where the two binaries are heavily mismatched, the dynamics of the seed particle may result in an oscillating compositional profile.

## Introduction

Multicomponent III–V materials in the form of nanowires offer some unique benefits: The nanowire shape enables the lateral relaxation of strain, meaning that nanowire heterostructures of highly mismatched III–V materials can be formed without the formation of defects along the interface which would occur for planar growth of the same heterostructure.^1^ In addition, grown nanowires can exhibit both the commonly stable zinc blende crystal structure as well as the metastable wurtzite structure,^2,3^ which can, for instance, change the band gap from indirect to direct.^4^ This system can thus provide possibilities of bandgap engineering which are not accessible using planar technologies, which can then be used either for fundamental physics studies^5^ or for optoelectronic applications.^6^

Nanowires are commonly grown using the vapor–liquid–solid (VLS) method. There, the solid nanowire grows from a liquid seed particle. The diameter of the seed particle is typically in the order of tens of nanometers, and the most well used material for the seed particle is Au which then alloys with the growth species. The seed particle is continuously supplied with growth species from a surrounding vapor phase. It is well established that the nanowire growth typically occurs in a cyclical layer-by-layer fashion, with repeating periods of incubation (where no solidification occurs, ending with a nucleation event), depletion (where the excess growth material in the seed particle is rapidly solidified after nucleation), and layer propagation (where the stable nuclei grow into complete layers).^7,8^

When modeling ternary nanowire growth, a constant composition in the seed particle is conventionally used, and this liquid composition is then used to find the composition of the layer by either equating it to the nucleation composition,^9^ finding the relative attachment rate of the two binaries at some nucleus size,^10^ or assuming it to be the same as the liquid composition.^11^ However, because of the small volume of the seed particle, the composition of the particle varies throughout the growth cycle, with a relatively higher supersaturation just before nucleation and a relatively lower supersaturation during layer propagation.^12^ It is commonly the case for nanowire growth that the seed particle consists of large fractions of Au and some of the growth species (e.g., In, Ga, Sb),^8,13^ and limited fractions of other growth species (e.g., As, P, N).^14^ Because of this, the variations in seed particle composition may asymmetrically affect different growth species. Thus, the cyclical nature of nanowire growth suggests that a theoretical approach which covers the growth of the entire layer would be desirable to accurately model the nanowire composition.

A common topic for discussing the growth of ternary III–V nanowires, using InGaAs as a model system, is the miscibility gap. The miscibility gap exists for the bulk system,^15^ and because of this, it is a naturally emerging result of many thermodynamic models for predicting the nanowire composition.^9,16,17^ However, experimentally grown nanowires have shown compositions within the miscibility gap.^11,18,19^ This calls into question what suppresses the miscibility gap in experimental growth, and one potential candidate would be strain. For bulk systems consisting of (pseudo)binary materials with a large mismatch (like the InAs–GaAs system), strain can lead to narrowing of the miscibility gap and prevention of spinodal decomposition in these materials systems.^20,21^ However, strain is difficult to capture in conventional steady state models, since strain has no effect if the composition of the solid is always constant. There are models based on a static liquid composition and a dynamic solid composition, which have been used to evaluate the effect of strain on the heterostructure interface.^22^ While this can be used to assess heterostructure interfaces, a static liquid composition neglects the cyclical nature of the growth and can thus never be used to understand how strain can affect the growth of a single layer.

In our previous Monte Carlo simulation framework,^23,24^ both the cyclical nature and growth history are included in the models, making it a suitable candidate for modeling ternary nanowire growth. There, we used classical nucleation theory as the tool for addressing the solidification process. For studying polytypism, this was deemed a reasonable approach, as the nucleation is expected to solely determine the crystal structure of the entire layer and a wurtzite nucleus and a zinc blende nucleus can be treated independently. For Monte Carlo simulations of ternary nanowire growth, a different approach was deemed necessary, both in order to capture the evolution of the composition throughout the entire layer and also to capture the interdependence of ternary nuclei of different compositions for treating the nucleation events. Two nuclei, one consisting of 10 InAs pairs and one consisting of 9 InAs pairs and 1 GaAs pair, should not be treated independently, as they differ only with the exchange of a single III–V pair. Thus, a treatment wherein this interdependence is captured is desirable.

To achieve our goal, we adapt our Monte Carlo framework to use a monomer approach to describe the solidification process instead of the full classical nucleation theory. The monomer approach remains classical in its treatment of the solidification process, meaning a nucleus grows or shrinks one III–V pair at a time according to the Becker–Döring rules. This captures the nucleation processes in this interdependent system but also allows the same framework to capture both the nucleation process as well as the layer propagation. To match this monomer approach in the solidification, we also use an atomistic view for the condensation and evaporation processes of the growth species. In addition, we incorporate strain energy and etching rules as optional settings, which can be added on top of a base model, to allow for separately evaluating their respective implication on the growth.

## Simulation Method

**Figure 1:**
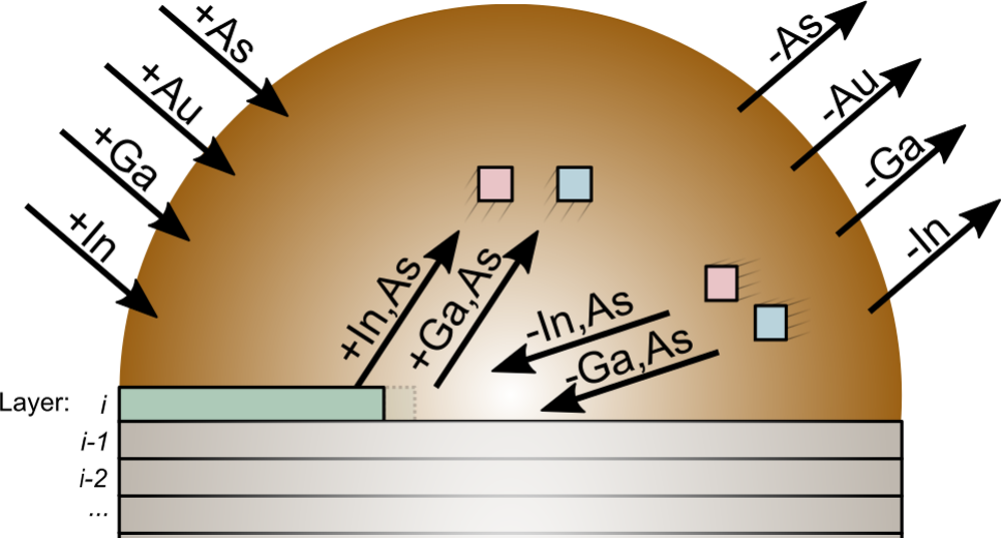
Schematic image of a seed particle and its nanowire during growth. The arrows represent the 12 events through which the seed particle can change over time, and the layer labeled *i*, colored green, represents the active layer with which the seed particle can exchange III–V pairs.

We chose to model the system from the perspective of the seed particle, as the axial growth of a nanowire can be thought of as a chain of events happening to the seed particle. The quaternary seed particle is comprised of a certain number of liquid atoms and is in contact with the surrounding vapor, which acts as a material supply to the seed particle, and the top of the nanowire, which acts as a material sink to the seed particle. We assume that the system can change via 1 of 12 possible events; there are 4 possible atoms (meaning either an As, Au, Ga, or In atom) which can condense onto the seed particle, 4 possible atoms which can evaporate from the particle, 2 possible III–V pairs (meaning either GaAs or InAs) which can be removed from the seed and attach to the solid nanowire, or 2 possible III–V pairs which can be added to the seed after detaching from the nanowire. This is shown in the schematic in Figure 1. From this perspective, the growth process of ternary nanowires can be modeled as if it is a seed particle performing a random walk in this six-dimensional compositional space. The history of this random walk reveals the composition of the solid nanowire which has been grown.

### Outcome Selection

To model nanowire growth in this way, a method is required for deciding which the next event will be and when it takes place. This cannot be chosen in a deterministic manner, but all different events can be assigned a probability which depends on the momentary individual rates, *J*_*i*_, of all events. The probability that a particular event *i* is the next event that will occur, *p*_*i*_, is calculated as^25^
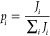
1

With a normalized probability for each outcome, which event will be the next one can be sampled using a random number, *R*_*U*_, from a continuous uniform distribution between 0 and 1, . This is done by finding the event *n* where , which will be the sampled event (with *p*_0_ = 0). Next, having all of the rates for the individual events also allows the generation of the time at which the next event occurs using a new random number *R*_*U*_:^25^
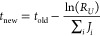
2

Having decided on which of the outcomes will happen, and when, the system can then be updated accordingly. If the outcome is, for instance, the impingement of an As atom, an As atom is added to the liquid seed particle, and if the outcome is the growth of InAs, one In atom and one As atom are removed from the liquid and one InAs pair is attached to the topmost layer of the nanowire. This topmost layer, meaning the layer with which the seed particle can exchange III–V pairs, is henceforth referred to as the active layer. When the active layer has successfully grown into a full layer, it becomes part of the nanowire layers and a new, empty active layer is created. The reverse case can also happen, where the active layer is empty and the decided outcome is to etch a III–V pair. The layer closest to the seed particle then becomes the active layer. After the update, the rates can be recalculated from the current state of the system, and a new event and time can be chosen until the desired simulation time has been reached.

### Rate Expressions

To complete the model, expressions for calculating each of the individual rates are needed. Starting with the liquid–vapor (LV) transitions, the impingement rate for species *i*, *J*_imp,*i*_, is calculated using the Hertz–Knudsen equation:
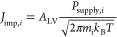
3

Here, *A*_LV_ is the area of the LV interface and *m*_*i*_ is the mass of the atom. *P*_supply,*i*_ is the supplied pressure of the species in the vapor phase, and this is one of the input parameters in the simulations, together with the temperature *T*. The evaporation rates are calculated analogously, using the same expression for the vapor pressure as was used in ref (24)
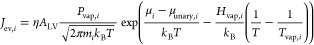
4

Here, η is an evaporation efficiency, set to 10^–4^, which is the same order of magnitude as was found to be a good fit in previous models.^24^*P*_vap,*i*_ is a reference vapor pressure of species *i* in its liquid phase at a reference temperature, *T*_vap,*i*_. *H*_vap,*i*_ is the enthalpy of vaporization for an atom of species *i*, and μ_unary,*i*_ and μ_*i*_ are the chemical potentials of species *i* in its unary liquid form and in the composition of the seed particle, respectively.

Next, we address the liquid–solid (LS) transitions. We calculate the rate at which a III–V pair of type *j* attaches to the active layer of the nanowire as
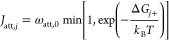
5

The pre-exponential factor ω_att,0_ represents the average frequency at which new pairs try to attach to the active layer. We approximate ω_att,0_ to be a set constant value of 1 GHz. Equation 5 bears much resemblance to the nucleation rate used in our previous simulation models,^23,24,26^ and there we have seen that changing the value and the dependencies of the pre-exponential factor has a negligible effect on the trends of the simulation. For this reason, we find this approximation reasonable. Next, Δ*G*_*j*+_ is the change in the total energy of the system due to attaching a III–V pair of type *j* to the active layer and removing the corresponding III and V atoms from the liquid. If the growth of *j* leads to a reduction in the energy of the system, then the attachment rate equals ω_att,0_, as indicated by the minimum function min[ ].

The detachment rate is given, analogously to the attachment rate, by
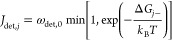
6

Here, we chose the same value of 1 GHz for the average frequency at which an existing pair attempts to detach from the active layer, ω_det ,0_, and Δ*G*_*j*–_ is the change in the energy of the system due to the detachment of a III–V pair of type *j*.

These rates all depend on what state the system is in, and thus require ways of calculating the necessary variables based on the current state, which will be covered next.

### Seed Properties

**Figure 2:**
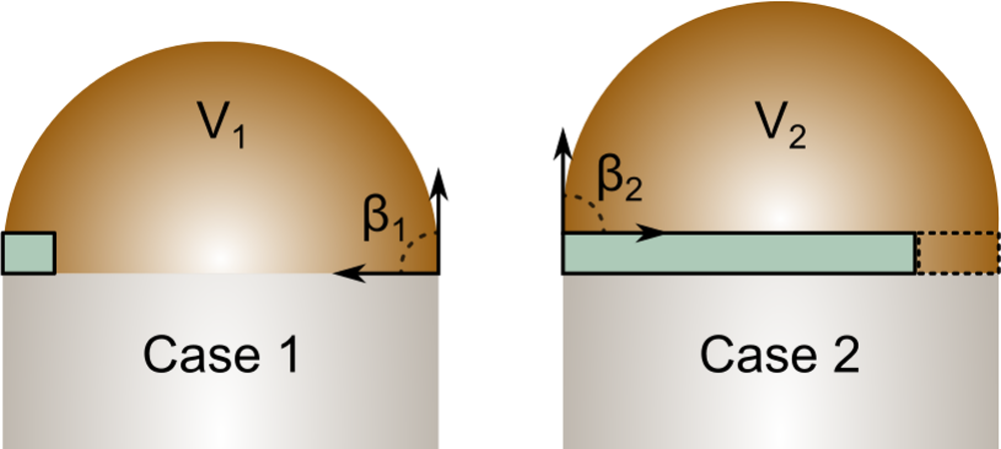
Illustration of the two geometries considered in the calculations of the area of the liquid–vapor interface of the seed particle. In case 1, the active layer is treated as being positioned inside the seed particle. In case 2, the active layer is treated as being positioned under the seed particle. *V*_*i*_ and β_*i*_ represent the volume and the contact angle of the seed particle, respectively. The dashed region in case 2 represents the part of the liquid seed particle which fills the “gap” in the active layer, meaning the part of the layer which has yet to be grown.

In each cycle of the simulation, the properties of the system need to be assessed. In the simulations, this starts by assessing the geometry of the system. As is discussed in ref (27), it is difficult to accurately assess the area and volume of the liquid particle when it contains a partially completed layer at the triple phase line. If the active layer contains a single III–V pair, then it is reasonable to approximate the seed as a spherical cap which contains the solid III–V pair. This spherical cap sits on top of the topmost completed layer of the nanowire, i.e., below the active layer, and the area of the liquid–vapor (LV) interface can then be adjusted based on the area of the vapor–solid (VS) interface of the active layer. The geometries explained are shown in Figure 2, and this case is labeled as 1. However, if one considers that the active layer has grown almost to completion, then the liquid would be displaced by this active layer, representing case 2. Then, it is more reasonable to consider the seed sitting on top of the active layer, with a small part of the liquid being removed to fill the gap in the hole which is shown as a dashed region in Figure 2. The volumes of the seed for the two cases are calculated as
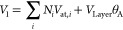
7
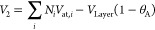
8

Here, *N*_*i*_ is the number of atoms of type *i* in the liquid and *V*_at,*i*_ is the volume of one such atom. *V*_Layer_ is the volume of a complete layer, and θ_A_ is the ratio between the area of the top facet of the active layer and the area of the top facet of a completed layer. For simplicity, we use a value for *V*_Layer_ which is independent of solid composition. The volume of the seed particle is then used to calculate its contact angle, β, using the assumption of the seed particle being a spherical cap. The volume of a spherical cap, *V*_Cap_, sitting on a nanowire with radius *R*, is given by^28^
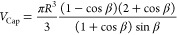
9

Next, we set *V*_Cap_ = *V*_1_ and use a fitted inverse function to find β_1_ and repeat the process using *V*_Cap_ = *V*_2_ to find β_2_. With the assumption of a spherical cap, a known radius and contact angle, *A*_LV_, can be calculated. However, the VS surface of the active layer also needs to be taken into consideration. Here, we use a simple approach. For case 1, we calculate the VS interface area of the active layer and subtract the same area from the liquid seed. For case 2, where the particle is ousted by the active layer, we add an additional LV area corresponding to the “gap” in the active layer, shown as a dashed region in Figure 2, which will be filled by the liquid. Combining this with the expression for the surface area of a spherical cap yields, for the two cases,
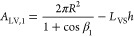
10
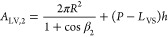
11

Here, *L*_VS_ is the length of the VS interface of the active layer, i.e., the part of the perimeter of the active layer which lies on the triple phase line. The height of a bilayer is given as *h*, and *P* is the length of the perimeter of a completed hexagonal layer of the nanowire. Finally, these two areas are used in a linear combination to calculate the final liquid–vapor interface area. The weight of each case depends on the fraction of the nanowire sidewall perimeter which is covered by the active layer, θ_*P*_ = *L*_VS_/*P*:
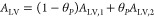
12

The choice of using θ_*P*_ as the weight between the two cases stems from the idea that, if the active layer would be ring-shaped and cover the entire triple phase line, then case 2 would be the appropriate representation. This also leads to a final expression which is self-consistent: The area of the seed particle is the same whether the nanowire consists of 9 layers and an active layer which has grown into a full layer or a nanowire with 10 layers and an “empty” active layer.

Next, the sidewall of the nanowire (excluding the active layer), *A*_VS,wire_, is calculated using the number of completed layers in the nanowire, *N*_layers_, and is given by
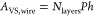
13

**Figure 3:**
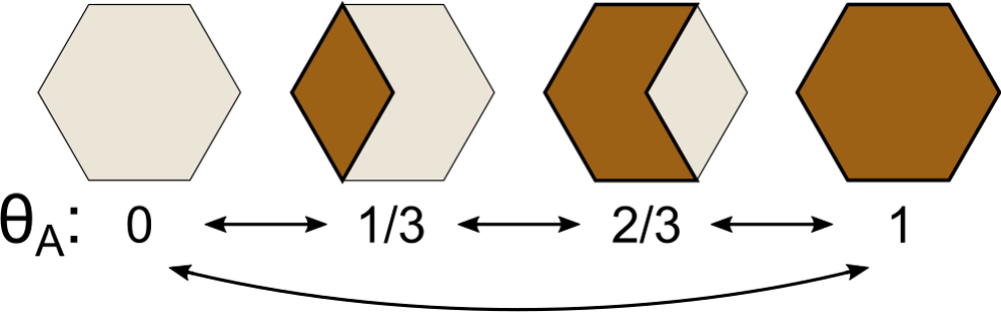
Schematic of the shape evolution of the active layer used in the model. The numbers represent the size of the active layer as a fraction of a complete layer, θ_A_.

Lastly, in the topic of geometry, the shape of the active layer is based on the work of Harmand et al.^7^ They found the initial shape of the active layer to be a rhombus and the terminal shape to be an “inverse rhombus”, as shown in Figure 3. We use an approximation of their results, wherein the shape is considered a rhombus when 0 < θ_A_ ≤ ^1^/_3_ and an inverse rhombus when ^2^/_3_ ≤ θ_A_ ≤ 1. This accurately captures the shape of the active layer during nucleation and also captures the negative differential surface energy of the active layer near completion. This negative differential surface energy will act as a “reverse nucleation barrier” if the model is used for simulating nanowire etching. Between these two shapes, we approximate the value for *L*_VS_ to be a linear combination between the rhombus at θ_A_ = ^1^/_3_ and the inverse rhombus at θ_A_ = ^2^/_3_. The length of the liquid–solid (LS) interface, *L*_LS_, is the same for both θ_A_ = ^1^/_3_ and θ_A_ = ^2^/_3_, and it is therefore kept constant for values ^1^/_3_ ≤ θ_A_ ≤ ^2^/_3_.

With the geometrical aspects covered, next the energies of the system are calculated. The total energy of the system has, in the base model, a bulk component and a surface component, as shown in eq 14. The bulk component consists of three parts; one relating to the liquid seed particle, one to the completed layers of the nanowire, and one relating to the active layer, as shown in eqs 15–17.
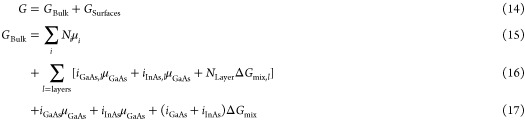


The first sum covers the chemical potentials of all of the atoms in the liquid phase. The chemical potential for each species, μ_*i*_, is calculated using the method described in ref (24) extended to include the In species. The In-related parameters used for the liquid phase can be found in ref (29). The second sum iterates over each layer in the solid nanowire, *l*, excluding the active layer. The chemical potential of each pair is summed up, and based on the solid composition of the layer, the total Gibbs free energy of mixing (*N*_Layer_Δ*G*_mix,*l*_) is evaluated.^15^ Lastly, the bulk energy of the active layer is evaluated in the same way as the completed layers in the nanowire, adjusted to the size (*i*_GaAs_ + *i*_InAs_) of the active layer. A term which will be used later in this work is the supersaturation of a III–V pair. We define this as the difference in chemical potential between a group III atom and a group V atom in the liquid phase and the III–V pair in the solid phase. For GaAs, this would be Δμ_GaAs_ = μ_As_ + μ_Ga_ – μ_GaAs_.

Analogous to the bulk component, the surface energies of the system also consist of three parts: one relating to the liquid seed particle, one to the completed layers of the nanowire, and one relating to the active layer. These expressions are shown in eqs 18–20.
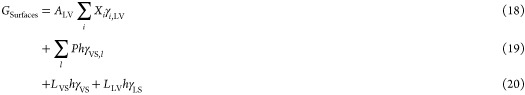


The LV surface energy is calculated using a linear interpolation between the liquid surface tension of each species. Values for surface tensions were chosen to be in line with reported values, extrapolated down to the temperature used in the simulations. These values were γ_Au,LV_ = 1.2 J/m^2^,^30^ γ_Ga,LV_ = 0.7 J/m^2^,^31^ and γ_In,LV_ = 0.5 J/m^2^.^30^ For As, no reference was found, and an in-between value of γ_As,LV_ = 1.0 J/m^2^ was arbitrarily chosen. Due to the low concentration of As in the seed particle,^8,23^ this choice should not influence the growth in any noticeable way.

For the sidewall surface energy of the solid phase, the lateral surface energy of each layer is calculated individually, with an area of *Ph* per layer. A linear combination between the (1 1 0) surface energy of GaAs and InAs was used for the ternary material. For the active layer, the areas for the VS and LS interfaces are given by *L*_VS_*h* and *L*_LS_*h*, respectively. For the VS surface energy, γ_VS,*j*_, a value of 1 J/m^2^ for *j* = GaAs and ^2^/_3_*J*/*m*^2^ for *j* = InAs was used. This is somewhere between the values in the literature which are based on broken bond calculations^32^ and those that are based on ab initio approaches^33^ while remaining close to reported ratios between the corresponding InAs and GaAs surface energies.^32^ The surface energies of the LS interface for the two III–V materials are generally unknown but estimated to be lower than the VS counterpart.^7,23,27^ For this reason, values of γ_LS_ = γ_VS_/2 were used. For InGaAs, a linear interpolation between the two binary sidewall energies was used.

### Time Optimization

The atomistic/monomer treatment used in this model resulted in a significant increase in the simulation time compared to our previously used ensemble approach.^23^ This has the implication that the simulation time does not scale well with the size of the system, meaning that simulating the growth of nanowires with radii larger than a few tens of nanometers is not plausible at this time. In addition, it was also deemed necessary to introduce an additional function into the model. The purpose of this function was to increase the speed at which the simulations could be performed, with a minimum effect on the results of the model.

Much of the computational time was found to be spent in the layer propagation periods of the growth. This is because the energy of the system changed very little by the attachment and detachment of III–V pairs in this regime. However, as has been shown for GaAs,^24^ layer propagation is generally limited by the impingement of As, and this imbalance where *J*_imp,As_ ≪ *J*_att,*j*_ and *J*_imp,As_ ≪ *J*_det,*j*_ led to an extremely high amount of LS events (meaning attachment and detachment events) before any As atom could be incorporated into the seed particle. This occurred around a shallow local minimum in the energy landscape, which did not progress the layer propagation nor did it typically change the composition of the active layer in a meaningful way. Thus, a function was added to decrease the LS rates during layer propagation. If at least three of these four LS rates were more than 10^3^*J*_imp,As_, then all LS rates were multiplied by a scaling factor. The scaling factor was set dynamically, such that the least likely of these events had a rate of 50*J*_imp,As_. This function was disabled during the nucleation (i.e., the layer contains less than 50 pairs) and layer completion (the layer requires less than 50 pairs to become complete).

### Strain and Etching Rules

In the model, we have chosen to include some optional settings, namely, strain and two separate etching rules. The reason for separating out these settings is that this allows the settings to be turned on or off at will, and thus, the isolated effect of each setting can be evaluated.

In the case of strain, this setting introduces a new set of energy terms, which results in an additional term in eq 14. For simplicity, we chose to treat each layer in the nanowire, including the active layer, as a coherently strained slab sitting on a semi-infinite “substrate: comprised of the layers beneath it. The energy stored in layer *l* is given by^1^
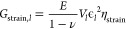
21

Here, *E*/(1 – ν) is taken as 150 GPa,^21^ where *E* is Young’s modulus and ν is Poisson’s ratio. *V*_*l*_ is the volume of the strained (active) layer, and ϵ_*l*_ is the misfit between the *l*th layer and its “substrate”. To calculate the misfit, a pseudocomposition of the “substrate”, *x*_sub,*l*_, needs to be approximated. Here, we assume that the layer *l* is affected most strongly by the layer just beneath it, *l* – 1, and that layer *l* is unaffected by the layers far away from it. We implement this by weighting each layer beneath *l* with a diminishing strength, which is halved for each layer (meaning half of layer *l* – 1, a quarter of layer *l* – 2, and so on). As the nanowire is finite, we use a cutoff distance of 8 layers, meaning that the pseudocomposition of layer *l* is unaffected by layer *l* – 9, and the strain energy of the first 8 layers of the stem of the nanowire is set to 0. This results in the following expression for the rest of the nanowire:
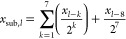
22

Here, *x*_*l*–*k*_ is the composition of the (*l* – *k*)th layer of the nanowire. The misfit for layer *l* is then based on the difference between the compositions *x*_sub,*l*_ and *x*_*l*_. We note here that other methods of calculating *x*_sub,*l*_ have been tested, such as simply using the composition of the layer beneath or averaging the composition of the part of the wire beneath the layer, with negligible effects to the trends which will be discussed here.

Finally, in eq 21, we introduced η_strain_ < 1 as a fitting parameter, used to reduce the contribution of the strain to the total energy of the system. Without this fitting parameter, the contribution of the strain was found to be dominant to such an extent that growth was completely suppressed. Treating each layer as coherently strained should represent an overestimation of the energy stored in the nanowire due to strain, making the use of the fitting parameter reasonable.

The next optional settings are part of what we refer to as etching rules. In the base model, any GaAs or InAs pair can freely be etched at any given time, regardless of where in the active layer that pair is placed. The etching rules are included to represent the idea that the etch rates should ideally be calculated for each III–V pair in the active layer individually and scale based on the surroundings of each pair. Removing a pair from the middle of the active layer should typically be much more energetically unfavorable than removing a pair at the growth front, and this could have implications on the composition if the relative incorporation rate of GaAs and InAs varies over the propagation of the layer. Incorporating this aspect into the framework would however require one to keep track of the position of each III–V pair in the entire nanowire, which is beyond the scope of this study. In the simulation, we instead approximate this effect in one of two ways as general etching rules. One is referred to as the propagation etching rule (PER), wherein only the last added pair is allowed to be etched away. This is a “last in, first out” rule, meaning that only the III–V pair which was added to the active layer last, can be etched. At any given time, the III–V pair which was not the “last in” has its etch rate set to 0 s^–1^. This is used because the last added III–V pair is by definition always at the growth front. The second etching rule is referred to as the layer etching rule (LER), wherein a completed layer is prevented from being etched. In reality, it is reasonable to assume that the etching preferentially occurs at one of the corner positions of the completed layer and propagates from there, which again requires the position of each III–V pair in the nanowire to be known. It should be noted that these simple implementations should overestimate the effects they are attempting to represent. A growth front consists of at least one III–V pair, with how many depending on the size and shape of the active layer, and etching from a complete layer is perhaps more selective than without the LER but certainly not impossible. That being said, these optional settings should illustrate the general effects one could expect from using individual etching rates.

## Results and Discussion

**Figure 4:**
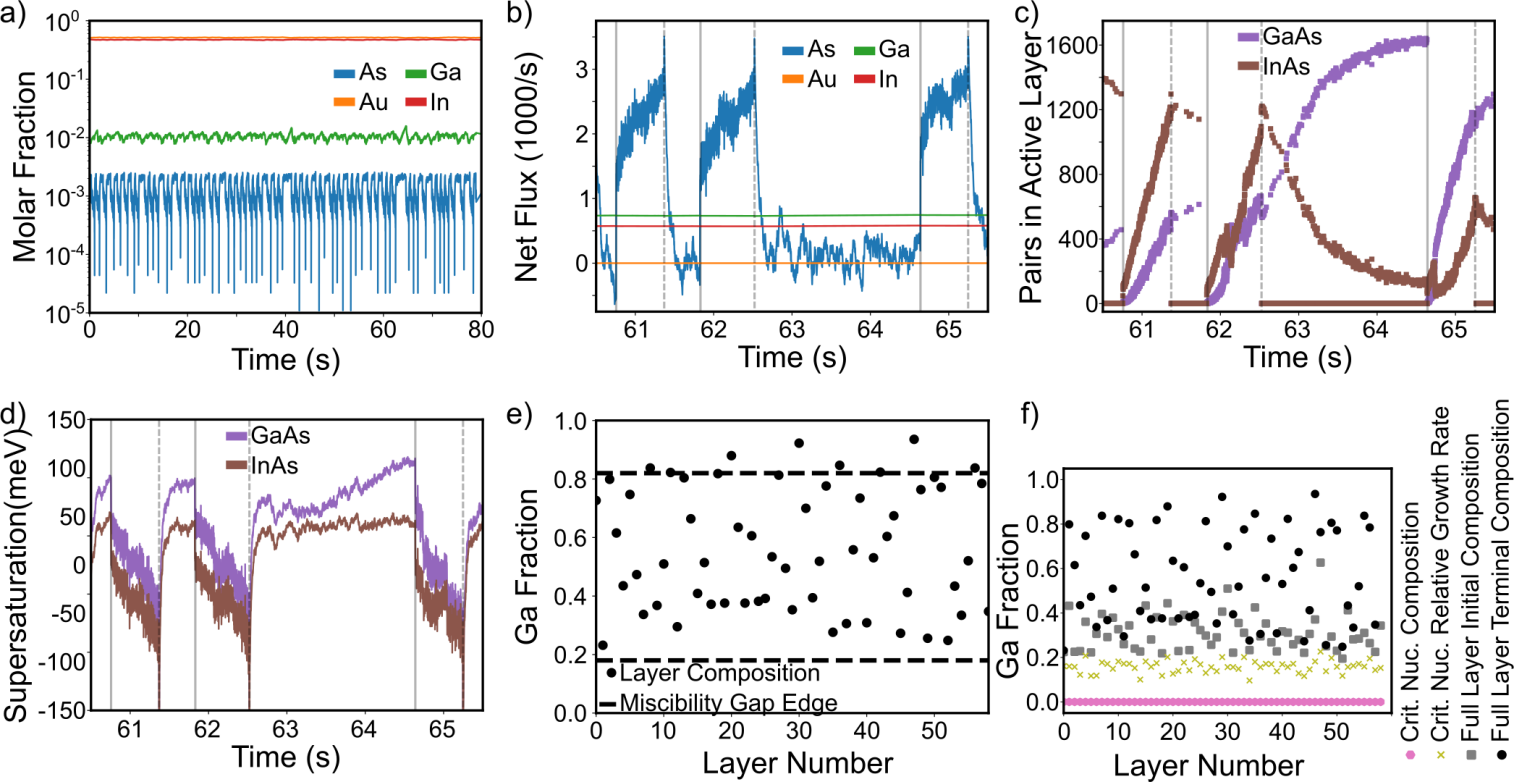
Results from the simulation using the base model. A graph showing the composition of the seed particle over the entire simulation (a). In parts b–d, graphs from the same 5 s period are shown, with the net condensation rate for each species (b), the number of GaAs and InAs pairs in the active layer (c), and the supersaturation for the binary III–V pairs (d). The vertical lines in these graphs represent a nucleation event (shaded line) or the start of the incubation period (dash-shaded). The solid composition for each simulated layer (together with the borders of the miscibility gap at the set temperature) is seen in part e, and a graph comparing the final layer composition to data from the corresponding nucleation event and data from the layer completion is seen in part f. Crit. nuc. composition refers to the composition of the critical nucleus, and crit. nuc. relative growth rate is the Ga/III ratio *J*_att,GaAs_/(*J*_att,GaAs_ + *J*_att,InAs_) for each critical nucleus. The full layer initial composition refers to the composition of the active layer when it first grows into a complete layer. The terminal composition is the composition of each layer after it has been overgrown by the next layer.

Here, the results from several simulations will be presented and discussed. While the input parameters for the model can be varied freely, for the purpose of this investigation, the model has been used to simulate growth where the supply pressures of the growth species were set to *P*_supply,As_ = 5*P*_supply,Ga_ = 5*P*_supply,In_ = 0.5 mPa and a temperature of *T* = 723 K was used. This placed the growth in the conventional regime where the V/III ratio is firmly above 1 and led to an average solid Ga/III ratio around 0.5. For each simulation, the starting composition was chosen by first performing an initial simulation. Once the initial simulation stabilized, the average composition for that simulation was then used as the starting composition, along with a starting contact angle of β = 90°. With these values, and a chosen nanowire radius of 10 nm, the initial state of the seed could be calculated. For the wire, a “stem” consisting of 15 layers with a Ga/III ratio of 0.5 was generated at the start of the simulations. The simulated results using these input parameters and the base model, i.e., without any of the optional settings, are shown in Figure 4.

Figure 4a shows the composition of the seed particle throughout the simulation, and from this, the general compositional hierarchy can be seen. The molar fraction of As in the seed particle oscillates greatly around 0.001, the molar fraction of Ga oscillates slightly around 0.01, and the In fraction is close to flat at around 0.45, meaning that there is an order of magnitude in difference between the three growth species. The liquid Ga/III ratio was found to be around 0.022 for this simulation, which is comparable to compositions found in thermodynamic models in the literature.^9^

In Figure 4b, the net impingement rates of the four constituents during a 5 s period are shown. The net condensation rate of Au was close to 0, as *P*_supply,Au_ was set to 0 and evaporation of Au was found to be highly unlikely at the temperature used. Similarly, the evaporation rates for In and Ga were also found to be close to 0; however, both of these species were supplied at a pressure of 0.1 mPa each. The difference in the net impingement rates for the two species stems from the difference in the atomic mass in eq 3. The lack of noticeable evaporation led to the completely static profile of the In and Ga net condensation rates, which differs significantly compared to that of As.

The As net condensation rate shifts between two regimes; the incubation regime where the net condensation rate oscillates around 0 and the layer propagation regime where there is a net positive impingement which increases until the active layer is fully grown. These regimes are visible in Figure 4c, where the numbers of InAs and GaAs pairs in the active layer are shown for the same period. This is consistent with As-limited layer propagation and group III limited nucleation,^24^ which is expected given the V/III ratio of the supply pressures. During the incubation periods, two curves can be seen in Figure 4c, one which is stationary near 0, meaning an empty active layer, and one which changes with time around the size of a full layer. This changing composition of a near-full active layer, which we will refer to as incubation drift, will be discussed in detail later. It should be noted that, while it was not evaluated specifically here, the simulation framework is capable of studying the potential effect that fluctuations in the As concentration (and as a result the net flow) may have on the nucleation.

The same periodicity is seen in the supersaturations for the two binaries in Figure 4d. By comparing Figure 4c and d, it can be seen that, during incubation, the supersaturations for both binaries are positive. After nucleation, the supersaturations first go toward zero but then turn negative during the growth of the final part of each layer. This is a result of the shape evolution used, which has a negative differential surface energy for the final inverse rhombus shape. This means that the total surface energy of the system actually reduces when growing the active layer, allowing for growth even at negative supersaturations. An additional observation is that the supersaturation of GaAs is, with very few exceptions, higher than that of InAs. This observation will be discussed more later.

The post-growth solid composition of the nanowire is shown in Figure 4e, together with the dashed lines which represent the boundaries of the miscibility gap at the set temperature. Here it is clear that the majority of the layers have a composition within the miscibility gap. Compositions within the gap are quite reasonable to achieve, considering the fluctuations in *X*_Ga_ seen in Figure 4a (which corresponds to fluctuations in the liquid Ga/III ratio) and the fact that the active layer is free to change its composition throughout the growth of the entire layer. Thus, these small fluctuations in the liquid composition can lead to large fluctuations in the relative growth rates of GaAs and InAs to the active layer. These fluctuations in the growth rates can be seen in the evolution of the active layer in Figure 4c.

In Figure 4f, data from the nucleation event is compared to the composition of the layer that grew from that event, to evaluate whether the nucleation can be a good indicator for the composition of the completed layer. For the nucleation data, the composition of the active layer at the moment of nucleation is included, as well as the relative attachment rate (meaning *J*_att,GaAs_/(*J*_att,GaAs_ + *J*_att,InAs_)) at the moment of nucleation. Since the simulation framework does not include nucleation as a separate event, the moment of nucleation is estimated through finding the first moment for each layer where the net growth rate for the active layer is positive. From Figure 4f, it is clear that the simulation framework shows no correlation between the composition of the completed layer and the composition of the active layer at nucleation, nor to the relative attachment rate during nucleation. Thus, the results of this simulation indicate that using nucleation to predict the ternary composition is not a suitable approach.

**Figure 5:**
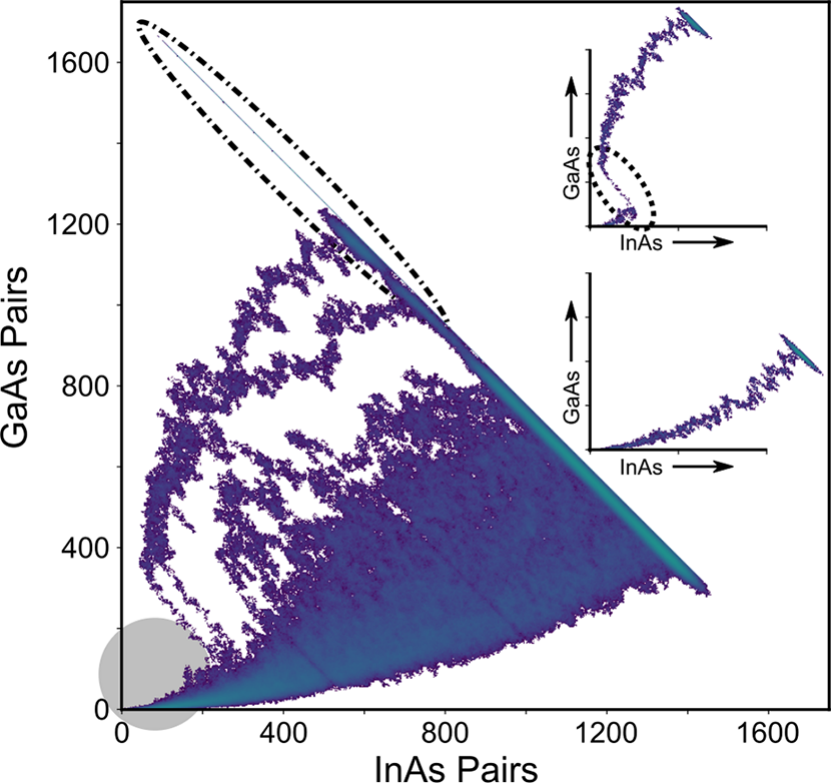
A heatmap of the compositional evolution of the active layer throughout the entire simulation. The two insets show the evolution of two individual layers in isolation, one which completed as a GaAs-rich layer (top) and one which completed as an InAs-rich layer (right). The shaded area highlights the regime where the nucleation events take place, the dashed ellipse in the inset highlights an In-rich to Ga-rich transition, and the dot-dashed ellipse highlights the region where the incubation drift is seen.

To understand this discrepancy between the nucleation events and the propagation, data from the evolution of the active layer is presented in the form of a heatmap in Figure 5. A white pixel in the heat map means that the active layer never experienced the corresponding combination of *j*_GaAs_ and *j*_InAs_. The more often a *j*_GaAs_ and *j*_InAs_ has been visited, the brighter the color in the heat map. It should be noted that there are “artifacts” showing as diagonal lines in the heatmap at layer sizes corresponding to ^1^/_3_ and ^2^/_3_ of a completed layer. These lines are a result of the change in the geometry of the active layer at these sizes.

From the heatmap, it is very clear that in the early stages of growth, which covers the nucleation of each layer, the active layer is always very In-rich. This is highlighted by the shaded area in Figure 5. This means that nucleation of the InGaAs layer preferentially occurs via a predominantly InAs-rich nucleus in the simulations. This is, albeit quite unexpected at first, a reasonable result of the materials system. As described previously, the γ_VS_ used for InAs is smaller than that for GaAs. This means that, in a situation where Δμ_GaAs_ = Δμ_InAs_, the nucleation barrier for InAs is smaller than that for GaAs, and the nucleation preferentially occurs for InAs. For nucleation of GaAs to be possible, Δμ_GaAs_ must thus be higher than Δμ_InAs_. In Figure 4d, it is clear that, while the supersaturation for GaAs is higher than that of InAs, the difference is small. This is also reasonable considering the growth behavior for large crystals. As a generic crystal becomes larger, the surface/volume ratio becomes smaller, and the growth of that crystal becomes increasingly dependent on the supersaturation and decreasingly dependent on the surface energy.^10^ In this regime, for the attachment rates of GaAs and InAs to be similar, their supersaturations must also be similar. While a layer in the nanowire is not exactly large enough to be considered bulk-like, the negative differential surface energy of the growth of the final ^1^/_3_ of each layer should lead to a similar effect.

Then, the active layer either continues growing In-rich, or there is a crossover from In-rich to Ga-rich, highlighted by the dashed ellipse in Figure 5. These crossovers are typically diagonal, meaning that they occur through exchanging InAs pairs with GaAs pairs, and not through preferential growth of GaAs in the layer propagation. The observed crossover can be understood from a thermodynamic perspective through the energy of mixing in the As–Ga–In materials system, which gives rise to the miscibility gap. The solid compositions which represent the ends of the miscibility gap are the compositions at which there are local minima in the energy of mixing; however, between the two, there is a local maximum at solid Ga/III compositions of 0.5, which would act as a barrier when it comes to changing from an In-rich layer to a Ga-rich layer. Thus, after the initial In-rich part of the active layer has grown, there must be a relatively high supersaturation for GaAs (compared to InAs) to surpass the energy maximum found at a solid Ga/III composition of 0.5. When GaAs reaches a sufficiently high supersaturation and does manage to surpass the barrier, it then becomes energetically favorable to continue to exchange InAs for GaAs, striving toward the local minimum at the border of the miscibility gap. Once this minimum has been reached (or too much Ga has been removed from the liquid), growth continues as normal.

A last point of interest in Figure 5 is the incubation drift, which is highlighted in the dot-dashed ellipse. This is a result of individual III–V pairs being exchanged during the incubation period for the next layer. While the system waits for the next nucleation and there are no III–V pairs in the active layer, a III–V pair from the previously completed layer can be etched and then immediately another III–V pair can take its place. When such a III–V pair has been etched, GaAs and InAs pairs will both attach at the same rate in the simulation. This is due to both III–V pairs being supersaturated (which is the case during a majority of the incubation period) and there being a negative differential surface energy for the final ^1^/_3_ of the layer. Thus, any drift in the composition is a result of the difference in the detachment rates from the completed layer during incubation. As seen in the dot-dashed area, this drift can cause significant changes to the composition of a layer, before the next nucleation happens.

### Simulations Using Etching Rules

**Figure 6:**
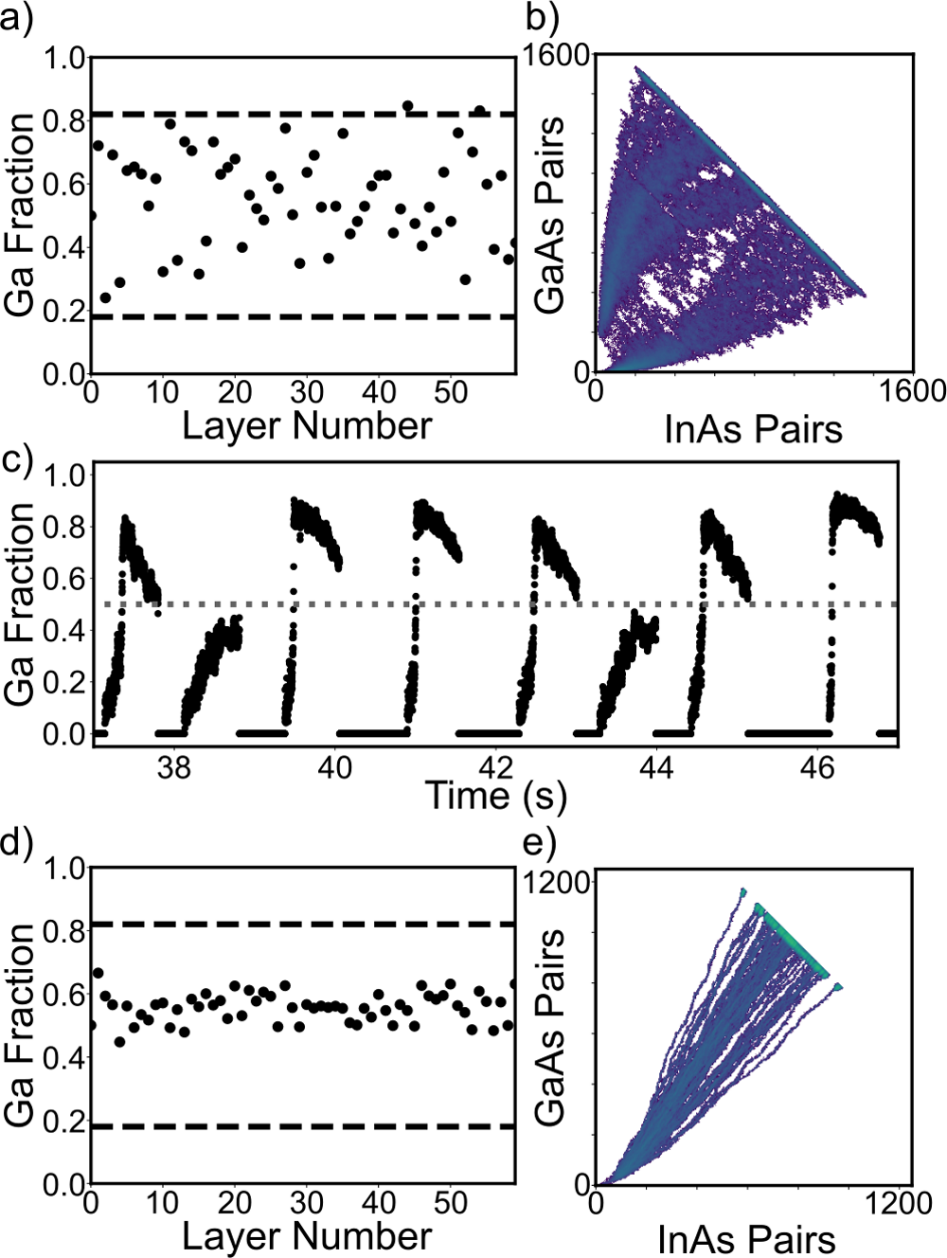
Results from two simulations with etching rules, either with LER (a–c) or PER (d, e). Graphs showing the solid composition of each solid layer (a, d) and heat maps of the evolution of the active layer (b, e). Graph showing the compositional evolution of the active layer during a 20 s period (c).

The base model shows that there are many factors which have been identified as contributing to the composition of the completed layer: fluctuations in the liquid composition, the mixing energy of the materials system, the preferential nucleation of InAs, and the incubation drift. We now turn to the optional settings in the simulation framework to further evaluate the impact of some of these effects. Before continuing with simulations which use optional settings, it is worth mentioning that the fluctuations in the solid composition of Figure 4e are substantial enough to assume that the results of the base model are not necessarily an accurate representation of reality. If these fluctuations were indeed found in experimentally grown InGaAs nanowires, with layer compositions regularly switching between In_0.25_Ga_0.75_As and In_0.75_Ga_0.25_As, this would likely be discernible in post-growth analysis using transmission electron microscopy. That being said, we next evaluate the effects of the two etching rules, keeping the same input parameters as those for the base model. The results from using a simulation using LER, wherein the incubation drift is suppressed, are shown in Figure 6a–c, and the results from a simulation using PER are shown in Figure 6d,e, which follows the “last in, first out” rule for etching.

Evaluating the composition of the nanowire in the LER case, Figure 6a, there is a slight but noticeable narrowing in the spread of the layer composition when the incubation drift is suppressed. However, the heat map of Figure 6b shows a comparatively much wider spread than the base model, which seems counterintuitive when considering that the layer compositions had a narrower spread. In the base model, if there was an above average *X*_Ga_ in the incubation periods, this would be balanced by a Ga depletion during the incubation drift. In this simulation, the LER meant that no such drift could occur, and as a result, the above average *X*_Ga_ is carried over to the next layer. In other words, the spread which previously manifested itself as an incubation drift is now instead occurring in the layer propagation.

The combination of these two observations, the narrower spread in the layer composition, and the wider spread during layer propagation can be understood by looking at the compositional evolution of the active layer over time, as is shown in Figure 6c. From this graph, it is clear that, whether the active layer is In-rich or has crossed over to Ga-rich, the evolution of the composition is toward a Ga/III ratio of 0.5. This suggests that the oscillations in the liquid composition and the crossover in the solid composition balance each other. A crossover from an In-rich active layer to a Ga-rich one depletes the droplet of Ga to such an extent that InAs is preferentially grown, leading to a narrowing in the layer composition distribution.

The results from the LER simulations differ significantly compared to the results from the PER simulations, which are seen in Figure 6d,e. From Figure 6d, it is clear that the distribution of the composition in the layers is very narrow, as is the spread in the heat map of Figure 6e. The use of PER effectively prevents the crossover effect, as the InAs-rich pairs which were attached early in the layer propagation cannot be exchanged for GaAs later on. This leads to a situation where the layer growth is reversible on a local scale, around the current composition, but effectively irreversible on a larger scale. Interestingly, if one only allows etching at the growth front, the simulation indicates that there is a high probability that the almost exact same composition will be regrown. This is seen in the strikingly small fluctuations seen in the pathways in Figure 6e. The solid composition then depends largely on the relative growth rate of GaAs and InAs whenever new As atoms are supplied to the seed particle.

### Simulations Using Strain

**Figure 7:**
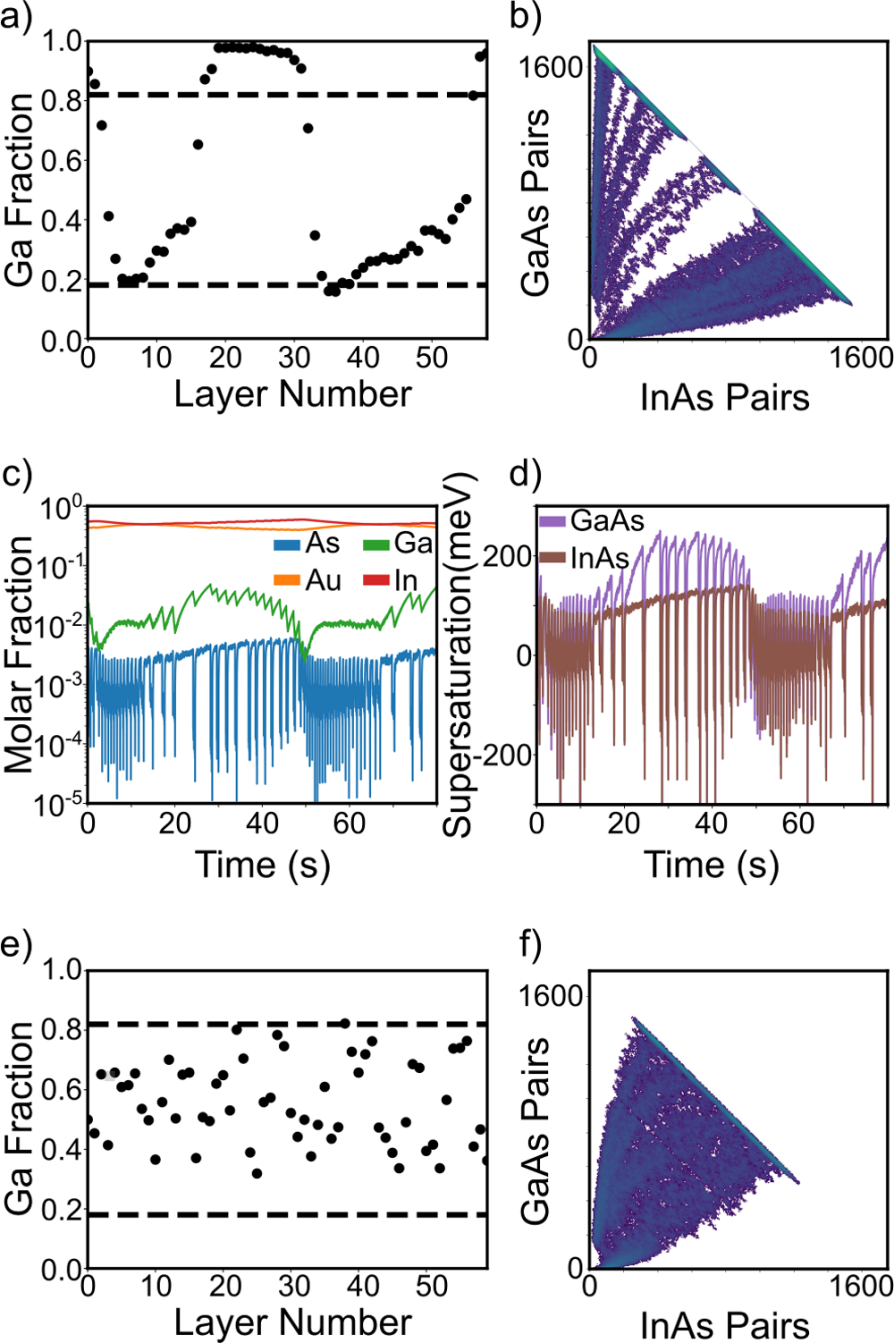
Results from two simulations which included strain, either with strong strain (a–d) or weak strain combined with LER (e, f). The layer composition is shown in parts a and e, and heat maps of the layer evolution are shown in parts b and f, together with the temporal evolution of the liquid composition (c) and the supersaturation for the two binary III–V’s (d).

Next, we evaluate whether the same narrowing of the composition distribution of the nanowire can be achieved by incorporating strain instead of using the PER. For this cause, two simulations with strain were performed, one which used the base model plus a “strong strain” (η_strain_ = 0.3) and one using the base model plus both LER and a “weak strain” (η_strain_ = 0.075). These results are presented in Figure 7.

Viewing the results of the strong strain case, it is clear that it did not have a narrowing effect on the layer composition in Figure 7a. Instead, large oscillations are observed where a long segment of very Ga-rich InGaAs is grown, followed by an In-rich segment and then a reversion to a Ga-rich regime. These segments are also reflected in the heat map of Figure 7b, where (apart from a few in-between layers) there is a distinct In-rich band and a distinct Ga-rich band.

While not in line with compositional analysis on experimentally grown InGaAs nanowires,^19^ it is nevertheless interesting to evaluate the reasons behind these results. This can be understood by considering the previous findings and the temporal data in Figure 7c,d. In this particular simulation, the first nucleation was relatively slow. Due to the compositional hierarchy (meaning *X*_Ga_ ≪ *X*_In_), the supersaturation of GaAs increases much more rapidly during incubation than that for InAs, leading to the next layer being Ga-rich. However, since nucleation was found to occur via an active layer which is very InAs-rich, having a Ga-rich top layer means that the nucleation barrier for InAs on top of that layer is increased compared to a more In-rich top layer due to strain. Thus, the next nucleation is also relatively slow, as a higher Δμ_InAs_ is needed, leading again to a more rapid increase in Δμ_GaAs_ and thus another Ga-rich layer. This continues until a tipping point is reached, where Δμ_InAs_ is sufficiently high to nucleate on the almost pure GaAs, which causes nucleation events in very quick succession. Since *X*_Ga_ is low, the Ga in the droplet is rapidly depleted and the grown layers become InAs-rich, leading to a lower nucleation barrier for InAs, leading to the rapid growth of several InAs layers. This continues until the droplet has been depleted of In to the point where Δμ_InAs_ is too low to nucleate on almost pure InAs. This leads to accumulation of Ga and the start of the next cycle.

These oscillations only occur when the strain is strong enough to have a significant impact on the In-rich nucleation, which is clear when viewing the results of the weak strain simulation in Figure 7e,f. With weaker strain, no oscillations can be seen, and the strain instead has a slight narrowing effect on the composition when compared to the corresponding case without the weak strain (Figure 6a and b). Thus, the simulations suggest that the effect of strain is non-monotonic and depends strongly on the strength of the strain energy. Going from the weakest strain to the strongest strain, the effect goes from nonexistent to a narrowing of the compositional distribution to inducing compositional oscillations.

### Discussion

From these results, it is clear that the simulation framework can provide different kinds of insights compared to conventional modeling techniques. The dynamics clearly become significantly more complex for ternary III–V nanowire growth compared to the binary counterpart, and this can give rise to many unexpected features during growth. Because all information from the simulations is readily available, interpreting these features becomes significantly easier compared to interpreting experimental results.

Considering the presented results as a collective, the nucleation events of new layers were always observed to occur via a very InAs-rich active layer, and this can be understood via its lower lateral surface energy compared to GaAs, as discussed previously. This has some interesting implications when it comes to the correlation between the flows of the growth species, the liquid composition, and the nucleation rate, as the nucleation rate solely depends on Δμ_InAs_. This means that the nucleation rate is thus independent of Δμ_GaAs_, and *X*_Ga_ only affects nucleation via the weak *X*_Ga_ dependence of μ_As_ and μ_In_. In a previous work, we showed that the composition of the seed particle (and thus *X*_Ga_) during the simulation of Au-seeded GaAs growth was determined by the flow of As.^23^ This means that the As flow dictated both *X*_As_ and *X*_Ga_, meaning that one can access most of the parameter space using a single parameter (at a given temperature) and varying the As flow has been used experimentally to, for instance, switch between growing high quality wurtzite and growing high quality zinc blende.^34^ Our results here suggest that the same would not be applicable for ternary growth. Because nucleation would be InAs-rich, the flow of As would only determine *X*_As_ and *X*_In_. The composition of the nanowire is sensitive to *X*_Ga_, and due to mass balance considerations, *X*_Ga_ would be determined by the Ga/III ratio of the net impingement rates. The final implications of this is that the composition of the seed particle during GaAs growth can to a large extent be tuned using the flow of As, whereas these results suggest that for InGaAs growth the seed particle composition is determined by the combination of the As, Ga, and In flows.

Next, it is of interest to discuss what the simulations show regarding the miscibility gap, as all of the simulations provide some insights into the subject. Most of the results show that, although the compositions of most layers were well within the miscibility gap, the growth can still be affected by the gap (or perhaps, more specifically, the profile of the mixing energy of the two binaries). As is expected for nanowire growth, we see fluctuations in the composition of the seed particle for all simulations. These fluctuations were mostly random, except for the case with strong strain where the fluctuations were periodic. The simulations were performed around an average solid Ga/III ratio of 0.5, meaning within the miscibility gap. As a result of this, these fluctuations have a large effect on the thermodynamics of the system and the layer composition which is thermodynamically favorable to form. This is clearly represented in the scattering of the layer compositions in all cases except one, when PER was enabled.

From all simulations, the nanowire composition profile, which best matched the expectations based on experimentally grown InGaAs nanowires, was the one simulated using the PER. This would suggest that both growth and etching occur at the growth front which, as clearly shown by the results of this investigation, has a significant impact on the growth behavior. PER led to solid compositions with a very narrow distribution, both throughout the growth of each layer and also between layers.

## Conclusion

In conclusion, we have presented a simulation framework which is capable of simulating the growth of Au-seeded ternary nanowires, which combines kinetic Monte Carlo with the addition and removal of individual species or III–V pairs. The simulation framework allows for the evaluation of the cyclical dynamics of nanowire growth, the impact of random fluctuations, and long-range effects such as strain, which are difficult to assess using more conventional techniques. The framework can be used for simulating the growth of binary, ternary, or other higher order III–V nanowires and would also be suitable for studying doping incorporation.

We have used the model to gain new insights into the complex dynamical aspects which could occur when growing InGaAs nanowires using Au as the seed material. We observe a disconnection between the solid composition during nucleation, which is found to be very In-rich, and the composition of the completed layer. We have shown how some strain can lead to further narrowing in the distribution of the layer composition, whereas a too large strain could result in oscillations in the solid composition.

While some interesting aspects of the dynamics of the growth have been revealed, the results also raised some questions in regard to which extent the etching rules are prevalent during experimental growth and whether periodic compositional oscillation can be induced in heavily strained systems. It is clear that our results highlight the need for further studies, both experimental and theoretical, to answer the complex questions in this highly dynamic system.
